# Methods for Detection of Bioimpedance Variations in Resource Constrained Environments

**DOI:** 10.3390/s20051363

**Published:** 2020-03-02

**Authors:** Eiko Priidel, Paul Annus, Andrei Krivošei, Marek Rist, Raul Land, Mart Min, Olev Märtens

**Affiliations:** Thomas Johann Seebeck Department of Electronics, Tallinn University of Technology, 12616 Tallinn, Estonia

**Keywords:** bioimpedance, dynamic range, synchronous measurement, lock-in detection, differentiation, cardiovascular system, non-invasive measurements, wearable devices

## Abstract

Changes in a certain parameter are often a few magnitudes smaller than the base value of the parameter, specifying significant requirements for the dynamic range and noise levels of the measurement system. In case of electrical bioimpedance acquisition, the variations can be 1000 times smaller than the entire measured value. Synchronous or lock-in measurement of these variations is discussed in the current paper, and novel measurement solutions are presented. Proposed methods are simple and robust when compared to other applicable solutions. A common feature shared by all members of the group of the proposed solutions is differentiation. It is achieved by calculating the differences between synchronously acquired consecutive samples, with lock-in integration and analog differentiation. All these methods enable inherent separation of variations from the static component of the signal. The variable component of the bioimpedance can, thus, be acquired using the full available dynamic range of the apparatus for its detection. Additive disturbing signals and omnipresent wideband noise are considered and the method for their reduction is proposed.

## 1. Introduction

Assessments of the working of human cardiovascular system has been one of the key tasks of medicine. It requires sensing as any other data acquisition task. The first truly non-invasive pulse recorder was introduced and patented by German physiologist Karl von Vierordt in 1855 - a sphygmograph. A “modern” mercury sphygmomanometer was not developed until 1896 and works of Scipione Riva-Rocci [1]. The first truly automatic measurement and recording devices showed up only in the late 20th century. Yet, the mercury sphygmomanometer has not disappeared from the scene. One of the key wishes has been formulated by the Research Professor and Director of Sleep Apnea Center of Kyushu University Hospital, Shin-ichi Ando: “I would like to have a blood pressure (BP) meter that is worn like a light wristwatch without any sensible pressure. … If such data can be transferred directly and easily to an electronic medical chart and be combined with other data, it would be very useful for the treatment of the patient and any kind of clinical research” [2]. Better diagnostic means and methods are clearly needed for ubiquitous early detection of the BP and its variation. There are a number of devices for acquisition of the static value of the BP, including some wearables. Variations are typically neglected in these devices. Probably, the best known tonometry device, which analyses the actual pressure curve is SphygmoCor [3], extensively evaluated in Mayo Clinic (Rochester, MN, USA) and other places [4]. Unfortunately, the method is rather complicated and operator-dependent, and can be used only by well-trained personnel, making it unsuitable for unobtrusive wearable acquisition. There is also a verifiable link between the actual pressure variations and ballistocardiographic (BCG), and photoplethysmographic (PPG) signals presented in [5].

Authors of the present paper consider the electrical bioimpedance (EBI) sensing over the radial artery to be very promising competitive solution, and can back it up with years of research and development. Introduction to the complex topic of the EBI and its acquisition can be found in [6], and discussions on how the acquired EBI values can be related to the state of the cardiovascular system are comprehensively introduced in [7]. One of the earliest devices envisioned by the authors, which reached eventually medical certification for noninvasive acquisition of the EBI, is presented in [8]. It was used during the clinical research phase in the East-Tallinn Central Hospital (ITK) for over 100 human experiments. It has been validated against the invasive “golden standard” procedure. It has also been validated against the SphygmoCor device in the clinical experiments carried out in the ITK. The conclusion is that it is less operator-dependent than the SphygmoCor of AtCor Medical and does produce valid results reliably. EBI based assessment of central aortic pressure is not new, Herscovici and Roller published their groundbreaking research in 1980’s [9]. Measurement was accomplished with four conductive Velcro electrodes, which were used together with inflatable standard pressurized cuff. Impedance plethysmography was promising for the assessment of the mean arterial pressure, and the used calculations resulted in acceptable correlation between direct and indirect measurements. Rudolf A. Hatschek has patented a non-invasive blood pressure measuring device and method in 1994 [10]. According the claims, blood pressure can be determined by acquiring the varying pulsatile volume and a speed of the pulse wave. For volume measurement, the EBI is suggested in addition to other possible means. J. Sola et al. provided experimental proof of usefulness of the electrical impedance tomography (EIT) on descending aorta on thorax in 2011 [11]. Many researchers have experimented with the EBI for central pressure in the aorta (CAP) assessment since then. Unfortunately, it has not yet resulted in widespread use of the method or development of relevant instrumentation. One of the reasons lies in the complexity of the task, including the need for advanced electronics. Current paper will not discuss relationships between EBI and cardiovascular parameters. Instead it will focus on the instrumentation for the ubiquitous acquisition of the EBI. This will be discussed in the following sections of paper.

## 2. EBI Acquisition

The topic is complex, and this explains the short list of success stories and modest number of developed devices, unless novel cardiac pacemakers are concerned where the sheer power of the EBI measurement method is used extensively. Two large problem areas are inherent, first, to the EBI acquisition electrode system, and second, to the electronics of the EBI measurement device itself. The device must operate reliably in rather harsh conditions, whereas minute variations of the EBI, which carry valuable information regarding the state of the cardiovascular system, can be masked by large motion caused artifacts, and the dynamic diapason required from the tiny wearable is huge. Electrodes play here crucial role. As stated in [6]: “around 85% of the problems in EBI measurement can be associated with the electrodes.” To acquire the EBI, one must let the current flow through the tissue and gather the voltage difference caused by the said current flow. The electrode system is used for coupling with the body. Four options could be considered in general [12]: Two electrode systems, both monopolar and bipolar, three electrode monopolar configuration and the tetrapolar four electrode or Kelvin connection. The names of the systems may be somewhat confusing. In case of the two and three electrode systems, the distinction between monopolar and bipolar refers to sensitivity distribution. Monopolar system should have only one significant region of high sensitivity and bipolar consequently two ideally equally sensitive regions. Situation is much more complex in case of the four-electrode connection to the object. The current flows through two of them and the voltage is monitored with the aid of two remaining electrodes. Sensitivity distribution, due to reciprocity [13], is cumbersome as it has negative sensitivity areas, and depends on the matter under electrodes. Having said that, the Kelvin method, essentially measuring transfer impedance between pairs of electrodes, drastically reduces the impact of the electrode-skin interface impedance to the results.

**Figure 1:**
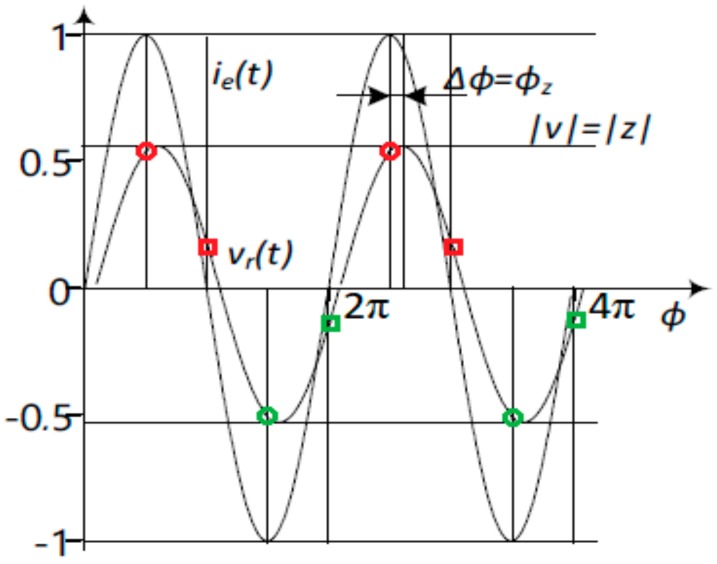
The unity-magnitude excitation current i_e_ (t) with a zero-value initial phase, and the voltage response V_r_ (t) with its magnitude, and phase shift. The normalized magnitudes of current and voltage in relative units given on vertical axis, and the phase on the horizontal axis. The samples related to the real part of the complex response of the i-th period s (i,1) and s (i,3) are designated by orange, and green circles, respectively, and samples corresponding to the imaginary part of the complex response of the i-th period s (i,2) and s (i,4) are designated by orange and green squares respectively.

In the following only four electrode measurement is discussed, and only the instrumentation aspects, not the electrodes and electrode systems themselves, which are discussed elsewhere. All of the instrumentation solutions are presented in this manner, even though they could be easily adapted for other configurations as well. The impedance is a complex ratio between the voltage on the object and the current through that object. Excitation with the voltage output source and current measurement can be used, or current excitation and voltage measurement applied. A generally arbitrary source is acceptable, if both the voltage and current are acquired during the measurement. For medical applications, current excitation prevails mostly for safety reasons, but it also minimizes the impact of the electrode impedances. Also, a sinusoidal excitation signal allows the simplification of the calculations, and the impedance is determined by the response voltage magnitude and phase (Figure 1), by taking advantage that sin(0°) = 0, cos(0°) = 1 and sin(90°) = 1 and cos(90°) = 0. Quadrature signal can be expressed in complex plane as vector with the length of M and angle φ where real and imaginary components can be expressed as $I=M\cdot \sin\left(\varphi \right)\;\mathrm{and}\;Q=M\cdot \cos\left(\varphi \right)$. As the modulus of the signal is $M=\sqrt{I^{2}+Q^{2}}$ we can see that on excitation signal phase angles 0° and 180° modulus equals to the imaginary part of the complex signal and on phase angles 90° and 270° modulus equals to the real part of the complex signal. The sampling, which is exactly on 0°, 90°, 180° and 270° phase angles, as seen in Figure 1, eliminates the need to calculate trigonometric functions in software resulting in lower CPU clock frequency and power consumption [14].

**Figure 2:**
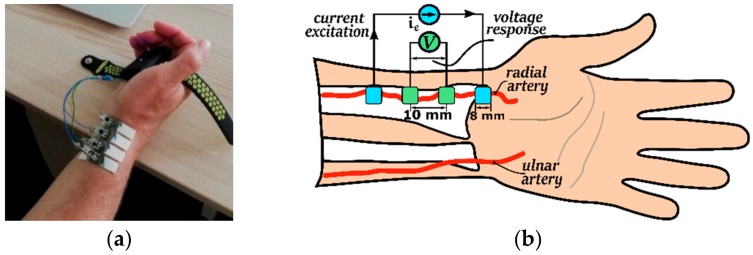
Electrodes (**b**) and their placement (**a**) during acquisition of the EBI.

**Figure 3:**
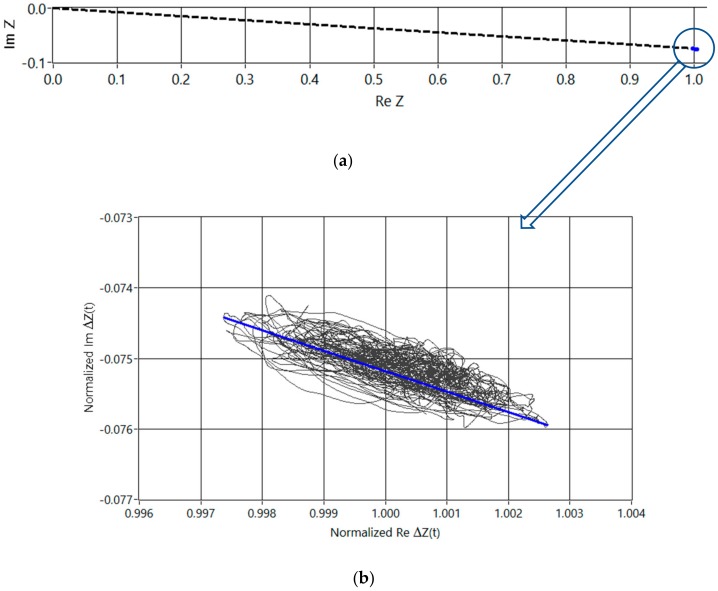
Normalized Nyquist plot of the impedance on the radial artery during several cardiac cycles. Real and imaginary parts of the base vector (dashed line, see (**a**)) together with modulation (blue dot at the end of the vector), and a zoomed-out modulation around the top of the base vector with average blue line (**b**).

Capacitively behaving bioimpedance is typically acquired in the range from kilohertz to hundreds of kilohertz. Therefore, EBI measurement on the radial artery (Figure 2 and Figure 3) is performed accordingly. As it can be seen slightly capacitive impedance has very modest variation. The biomodulation is caused mostly by pulsating blood flow.

Very small changes have to be acquired and this requires a wide dynamic range from the measurement apparatus. Abrupt changes in impedance of the contacts between the skin and the electrodes require close attention as well, otherwise the motion caused artifacts may saturate the measurement channel for a longer time and cause some slow signal wanderings afterwards.

## 3. Closer Inspection of the Measurement Situation

A set of measurements is conducted on the radial artery to illustrate the problem. Actual measurements were conducted multiple times with several different electrodes including gold plated printed circuit board (PCB) electrodes and standard electrocardiography (ECG) electrodes. The setup with the ECG electrodes is shown on Figure 2a. Placement of the electrodes in relation with the wrist and radial artery is further depicted on Figure 2b. Size, distance, location and the construction of the electrodes has been experimented with and refined during longer research period and described in different papers, such as [8] and [15]. In brief summary it can be said that the optimal setup for repeatable results consists of four equally spaced electrodes of 2–4 mm diameter roughly. The measurement results are normalized for two reasons. First, the results vary depending on the exact position and coupling of the electrodes. Secondly, the relationship between the base value and the periodic blood pulsation caused change is of interest here. The measurement results acquired from the radial artery are presented in Figure 3. Impedance values on real and imaginary axis are divided with average value of the real component as it is visible on the figure. Signal is taken from single measurement during several cardiac cycles on the radialis of volunteer. Normalization is done by dividing measurement results by center value of the samples. As seen from Figure 3 phase of the response signal is in the range of 4.27 to 4.33 degrees and Re(∆Z) variation is 0.52%. Base level of the bioimpedance is normally in the range of 100 Ω to 1 kΩ depending on electrode material, distance and location. Impedance base level and phase is similar between subjects – large scale study of over 220,000 persons in Germany [16] defines average impedance of the human body, which shows very similar phase angles to Figure 3, even though it is measured at different location and only at 50 kHz. The reference study of dielectrical properties of tissues [17] marks the magnitude of the variability of readings between different subjects. Impedance variation on the radialis caused by cardiac activity can be considered small, around 0.5%, compared to base impedance and is in the range of some hundreds of milliohms. Impedance measurements on the hand of the author on Figure 3 and comparison with measurement results from other works are shown in the Table 1. Variability of bioimpedance measures in normal adults discussed in detail in [18] shows also remarkably similar phase angles as in [16], but now at 100 kHz. Also, the changing part of the bioimpedance measured in [19] on the wrist falls very close to the results acquired when conducting measurement depicted on Figure 3.

A signal was acquired with a well-known Zurich Instrument, AG HF2LI 50 MHz Lock-in Amplifier, together with HF2TA Current Amplifier, to ensure the results are repeatable elsewhere. The gathered information was analyzed and presented in the custom made LabView-based environment. The measurements were conducted in a wider scale at frequencies 10 kHz, 25 kHz, 50 kHz and 120 kHz in different conditions. The measurements with frequency of 120 kHz had the best signal quality and showed less motion artefacts compared to lower frequency measurements. The results presented here are from the single measurement frequency of 120 kHz, as it was determined to be the most viable for future experiments, and with the author sitting in quiet resting position in the chair with armrests. Actual impedance values and their dependence from the electrodes and their placement is not the subject of the investigation here. Instead relationship between the base value of Z_0_ and magnitude of the change ∆Z(t) on top of it is emphasized:

Z(t) = Z_0_ + ∆Z(t), (1)

Question may rise regarding representativeness of Figure 3, since it is measured on a single person. It has been shown however by Camelia Gabriel in her reference work (together with colleagues from the Brooks Air Force Base (USA)) [17] that: “Biological tissues are inhomogeneous and show considerable variability in structure or composition and hence in dielectric properties. Such variations are natural and may be due to physiological processes or other functional requirements. The spread of values ranges from about ±5% above 100 MHz to ±15% at the lower end of the frequency scale.” Therefore, it is reasonable to assume that the variation is not larger than presented by C. Gabriel, and Figure 3, can be considered sufficient as it is only showing the scale of the problem and not exact values. Moreover, minimal change of the bioimpedance of 0.1% is assumed, which is very conservative compared to 0.5% in Figure 3 and is considered to have sufficient margin of five times compared to the worst-case deviation of ±15%, i.e., max 30%. In the following sections it is often called “small change” instead of actual number.

## 4. Two Devices for Comparison

**Figure 4:**
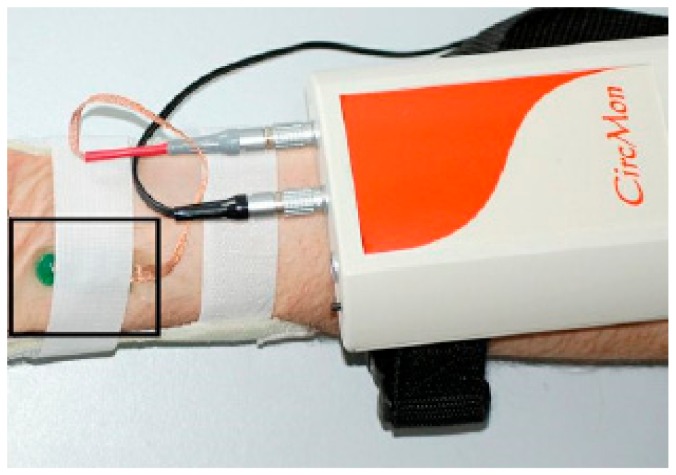
Noninvasive cardiovascular circulation monitor CircMon.

**Figure 5:**
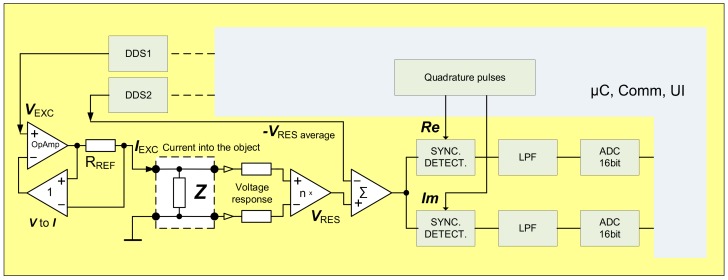
Block diagram of the EBI measurement device using direct carrier compensation method.

Several methods have been proposed to overcome these graphically presented difficulties. Simplest is capacitive AC decoupling in analogue domain. To minimize the distortion of the waveform of roughly 1 Hz signal (which is typical heart beat frequency) the corner frequency of the high pass filter (HPF) must be substantially lower, which results in large time constant. Even though the described approach works rather well, it has a serious drawback very slow reaction to the possible large changes of the input signal. Such changes can easily appear due to the movement of the system. Ideally the final solution should be free of that kind of lag. The second method is used for the acquisition of the EBI signal during the ITK experiments in the clinical device CircMon BT101 (JR Medical OY, Tallinn, Estonia) (Figure 4)—the direct carrier compensation (DCC) [20] (Figure 5), where DDS is Direct Digital Synthesizer, uC is microcontroller, Comm denotes communication interface, in current case Universal Serial Bus (USB) together with Bluetooth low energy (BLE), LPF is Low Pass Filter, ADC is Analog to Digital Converter, V_exc_ is excitation voltage, I_exc_ is excitation current, V_res_ is response voltage, R_ref_ is current determining reference resistor, and UI is User Interface. DCC, while working very well, requires usage of several synchronized oscillators, which complicates the schematic, requires rather complex adjustment software and ultimately results in larger, more expensive device with higher energy consumption than optimal. DCC is acceptable for the medical device used in the clinical surroundings, but unobtrusive and pervasive monitoring of the cardiovascular system would certainly benefit from more streamlined solution. Another example of the complex multifunctional device for laboratory use is given in [21]. Multi-frequency instrument for simultaneous multisite EBI acquisition is described. Essentially high-speed sample by sample compensation is used. Again, method is good but not suitable for wearable electronics.

**Figure 6:**
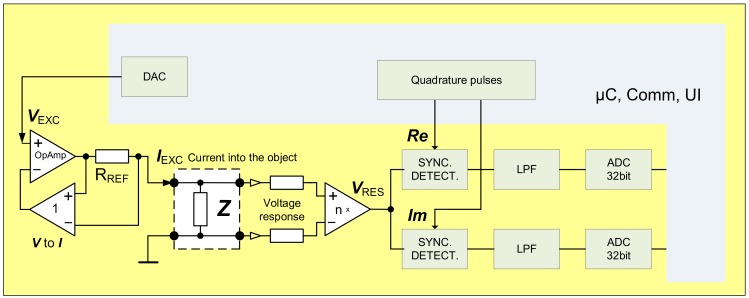
Main components of the “brute force device” using high resolution ADCs for detection of the EBI.

The second contender stepping up in current comparison between different methods was the “brute force device” with the best possible modern analog to digital converters (ADC) on board (Figure 6). Atmel AVR microcontroller XMEGA256, one of the latest 32-Bit Linear Technology Over-Sampling ADC’s the LTC2508-32 (Norwood, MA, USA), and the BLE Version 4.0 + radio module were used. Energy supply is from an on-board lithium-ion battery to minimize parasitic coupling issues. The ADC has reasonably low noise and low power (for more information please refer to the datasheet of the used ADC), as well as integrated configurable digital filter, which enables immediate suppression of the unwanted disturbances. Digital synchronous demodulation is problematic due to the slow output data rate (3.9 kSa/s) of the converter, and limited computing power of the used microcontroller, and therefore analogue synchronous demodulators were used instead, as in the previous case of DCC. Therefore, the detection occurred in the analogue domain without the compensation. When compared to the DCC method, the multiplication in the analogue domain with following direct conversion does not have roughly 6–8 effective bits added to the nominal resolution of the digitizer (ADC). It was amply compensated with the double-up bit count of the following ADC. Both, real and imaginary quadrature channels were used in the previously described devices. Careful examination of the Figure 3a would suggest that the imaginary channel might be omitted, either by compensating the tiny phase error of roughly four degrees, or even ignoring it altogether, because the error introduced is not significant in the current application. That is exactly the path taken in design of following three competing novel solutions.

## 5. Three Novel Solutions, Materials, Methods, Preliminary Results and Discussion

Three different methods for the separation of the base value of the impedance from the time varying biomodulation were proposed, examined, simulated, and to a various extent prototyped. A common feature shared by all members of the group of the proposed solutions is differentiation. First, realization [22] is based on the calculation of differences between synchronously acquired consecutive samples. It has been shown that it is possible to demodulate the signal with simple additions and subtractions when using quadrature samples shown also on Figure 1. It has been described in several papers, but the most comprehensive overview is given in [21]. It was stated that the direct current (DC) component can be calculated as:

(2) $$\mathrm{DC}\left(i\right)=\frac{s\left(i,1\right)+s\left(i,3\right)}{2}=\frac{s\left(i,2\right)+s\left(i,4\right)}{2}.$$

The real and imaginary parts of the complex response signal (V) expresses impedance here could be computed according to:

(3) $$R_{e}\left(V\left(i\right)\right)=\frac{s\left(i,1\right)-s\left(i,3\right)}{2}\;\mathrm{and}\;I_{m}\left(V\left(i\right)\right)=\frac{s\left(i,2\right)-s\left(i,4\right)}{2}.$$

And finally, the modulus and phase of the complex response signal V are found as:

(4) $$|V\left(i\right)|=\sqrt{{\left(R_{e}V\left(i\right)\right)}^{2}+{\left(I_{m}V\left(i\right)\right)}^{2}}\;\mathrm{and}\;\Phi \left(i\right)=\arctan\frac{I_{m}V\left(i\right)}{R_{e}V\left(i\right)}.$$

It is possible to decrease processing load and acquired samples even further by measuring only either positive or negative half period of I_m_V and using calculated DC component in I_m_V calculations:

ImV(i) = (s(i,2)) − DC or ImV(i) = (s(i,4)) − DC.(5)

In that case, only three measurements for the whole period are required in order to calculate the real and imaginary parts of the complex response signal.

In [21], the calculations were done in digital domain after conversion of the analog samples to their digital counterparts. These calculations can be done in analog domain when consecutive samples are held in separate sample and hold (SH) units, before they are finally quantized. The resulting R_e_V, I_m_V, and ∣V∣ contain the sum of the base value and the modulation on top both, in analog and digital domain calculations. It becomes more interesting if we consider the actual DC shift to be equal to zero, which is easily achievable by high pass filtering the high frequency response signal, then Equation (2) will have entirely different meaning. Now, the calculated sums show the difference between the values of the samples, corresponding to real and imaginary parts of different half periods, or in fact it is equal to ∆V(i) for either, real part or imaginary part for that period. ∆X(t)/∆t when ∆t is short enough can be viewed as derivative of the original response signal, hence the word differentiation in the description of the proposed three methods:

(6) $$R_{e}∆V\left(i\right)=\frac{s\left(i,1\right)+s\left(i,3\right)}{2}{\;\mathrm{and}\;I}_{m}∆V\left(i\right)=\frac{s\left(i,2\right)+s\left(i,4\right)}{2}.$$

**Figure 7:**
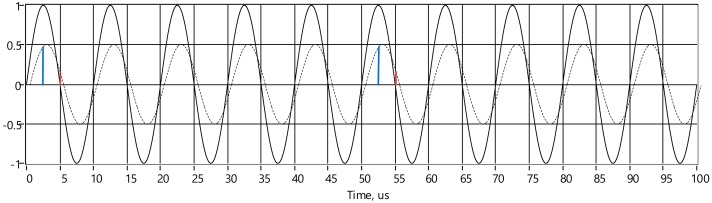
Down-sampling of the response signal using only positive half periods. Solid line is unity magnitude excitation current, dashed line is response voltage. Samples related to the R_e_V(i) and I_m_V(i) in blue color and red color respectively.

Removing DC shift simplifies sampling even further, in order to calculate response signal real and imaginary parts, only two samples per whole period is required. The differences can be calculated by using only samples belonging to either positive or negative half periods (Figure 7):

(7) $$R_{e}∆V\left(i\right)=s\left(i,1\right){\;\mathrm{and}\;I}_{m}∆V\left(i\right)=s\left(i,2\right).$$

These differences can be calculated in many different ways leading to different realizations of the circuit. For example, prolonging the distance between two samples for integer number of whole periods can be considered, effectively resulting in slower sampling. Such an under-sampling may be beneficial for reduction of the energy required for sampling, and also when the sampling process has physical limitations due to cheaper components. The under-sampling rate is limited by the spectrum properties of the response signal. In usual bioimpedance measurement applications, the signal spectrum characteristics are related to the cardiovascular processes where signal spectral components are in kilohertz range. The under-sampling rate should satisfy Nyquist criteria and sampling rates over 2 kilo-samples per second are commonly sufficient. Compared to excitation signal frequency, sampling rates can be tens of times slower, which optimizes energy consumption and reduces the required computational power. Additionally excitation signal can be interrupted between sequential sampling events that reduces power consumption even further. Obviously, transient processes must be taken into account between re-enabling excitation signal and acquiring next sample.

**Figure 8:**
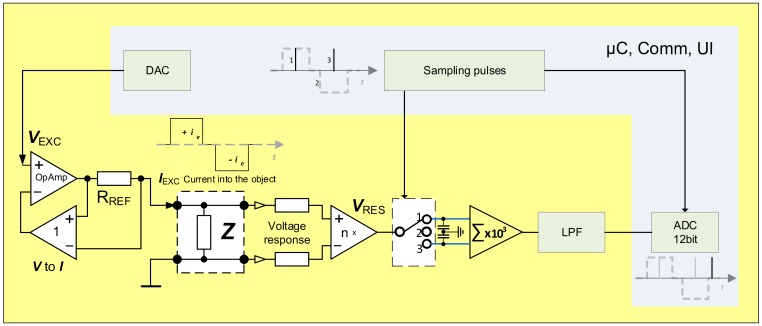
Simplified representation of the EBI measurement device with derivative sampling.

Device corresponding to first half of the Equation (6) is shown on Figure 8, where *I_EXC_* (+*i_e_* and −*i_e_*) is excitation current, *V_RES_* is response voltage, DAC is Analog to Digital converter for excitation signal generation.

**Figure 9:**
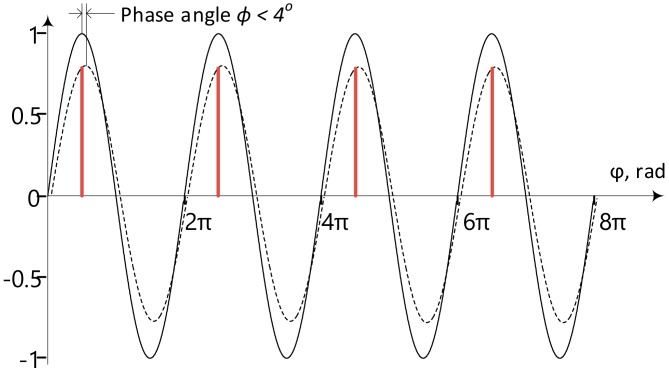
Sampling $|V\left(i\right)|$ in case of small phase angle of the impedance vector using only positive half periods. Solid line is unity magnitude excitation current, dashed line is response voltage. Samples related to the R_e_V(i) are in red color.

When proposing this realization, it is assumed that the phase angle of the impedance vector (shown on Figure 3a) is typically less than 4 degrees in case of excitation signal frequency of 120 kHz, and R_e_V(i) can be taken approximately equal to∣V(i)∣, without introducing large errors, at the same time considerably simplifying the physical realization of the device, (Figure 9).

Maximum error introduced by discarding imaginary part of the response can be found as ratio of the modulus of the complex response signal to the real part.

Modulus of the complex response signal V is:

(8) $$|V|=\sqrt{I^{2}+Q^{2}}.$$

As real and imaginary components of the complex signal are:

(9) $$I=R_{e}V=\left|V\right|\cdot \sin\left(\varphi \right)\;\mathrm{and}\;Q=I_{m}V=\left|V\right|\cdot \cos\left(\varphi \right).$$

It can be shown that:

(10) $$\frac{V}{R_{e}V}=\sqrt{1+{\left(\tan\left(\varphi \right)\right)}^{2}.}$$

**Figure 10:**
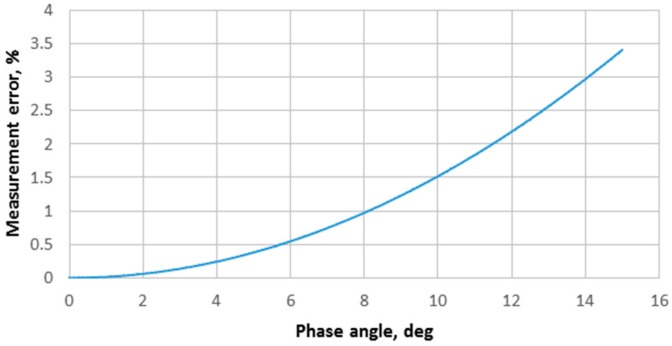
Measurement error compared to phase angle of the impedance vector.

Estimated measurement error introduced by sampling only R_e_V in that case based on Equation (10) is approximately 0.24% (Figure 10). Actual response can also be easily calculated as:

(11) $$|V|=\sqrt{R_{e}V^{2}.}$$

**Figure 11:**
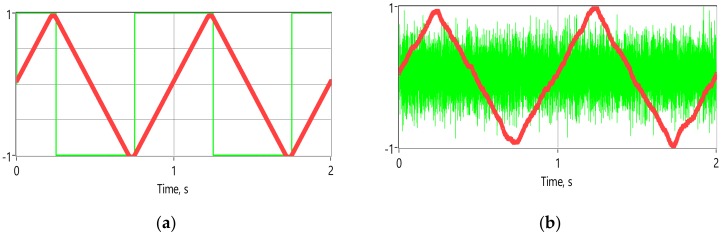
Normalized results of the differential sampling solution: without added noise (**a**), and the same signals with 0.1 mV noise added (typical situation during EBI acquisition) (**b**); all the curves are normalized for better readability.

Actual R_e_V(i) can be easily measured and calculated (3) and gives very good indications regarding the quality of the electrode attachment to the body. Why is the calculation of the R_e_∆V(i) more beneficial than direct calculation of the R_e_V(i)? There is no real benefit, if the calculations are done entirely in digital domain. If the difference between consecutive samples is calculated in analog domain, things change considerably. Now, we need to digitize only tiny variations separately from large base vale. Furthermore, these tiny variations are randomly around common mean, which is determined mainly by the omnipresent noise. It means that relatively slow variations in measured impedance caused by the biological processes, at least when compared to the fast-changing excitation signal, will not show up. Even large artifacts will disappear due to tiny change in short time window. Method has been extensively simulated in the LabView environment with convincingly good results [22] (Figure 11). Derivative sampling circuits can be made from discreet components, but more importantly they are easily integrated when purpose-made silicon is designed. As it is seen on the Figure 8 the differential sampling solution performs well, with or without an additive noise, even if only 12-bit converter is used. The12-bit resolution was chosen for comparison, because many microcontrollers and digital signal processors (DSP) have similar ADC’s.

In Figure 11, a carrier of 1 V amplitude is modulated by 0.1 mV and 1 Hz triangle wave. Normalized results are depicted as follows: A narrow green line square wave (normalized to unity magnitude) represents the calculated differences between adjacent samples, a bold red is normalized result of the 12-bit conversion of the differences integrated afterwards digitally. Note that the actual impedance modulation signal is fully restored, even while the derivative square wave has totally disappeared into the noise on Figure 11b.

There is also related patent application in process [23], which considers also an extension to the described method. The general sampling process acquires the value of the original signal plus some additive noise at the sampling point. Mathematically, the sampling instance is considered infinitely short in time. Immediately, it appears impossible to acquire samples in the described manner in real life. Instead of finite length sampling instances are present, typically very short, compared to the period of the maximum frequency component of the signal to be sampled, and at least twice faster than mentioned period according to well-known Wittaker-Kotelnikov-Shannon (WKS) sampling theorem. Naturally, the question may arise of what happens if we prolong the sampling instance? Real samplers obviously require some time to take the sample and settle afterwards, and therefore, have so-called aperture errors [24]. The sampler integrates the incoming signal to be sampled short while during, and after, the sampling pulse, as the sampling switch cannot change its state instantly. Generally, these aperture errors are considered harmful and should be minimized. From the other side, this integration can be viewed as filtering operation, which could help to improve the achievable signal to noise ratio (SNR).

**Figure 12:**
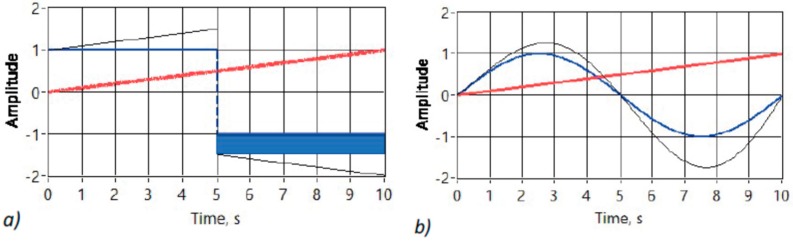
Synchronously integrating AM detection, when the carrier is square wave (**a**), and when the carrier is sinusoidal (**b**). Carrier is modulated in both cases with linearly growing ramp. Unmodulated carrier is wider blue line, modulated carrier is narrow black line, and modulating signal wide red.

That, in turn, brought up the idea of further investigating the performance of the system, when infinitely short samples are replaced with half-period integrals instead. Here, the answer is considered only in limited case of synchronous sampling, which is locked-in to the periodic excitation signal [25].The basic principle can be seen on Figure 12. Modulation with linear ramp a∙t, where a is arbitrary constant and t is time, is considered for simplicity of the discussion.

That simplification seems to be valid, if we consider very slowly varying biomodulation (few Hz, with relevant bandwidth of the signal up to some hundred Hz) and compare it with relatively fast excitation (120 kHz excitation in current case of the EBI acquisition) signal. The case of the square wave carrier is easier to comprehend visually. As it can be seen in the Figure, the net integral over the full carrier period is zero, if the carrier is not changing during that time. Yet, one of the half periods has higher magnitude than the other when modulation of the carrier is present. A dark rectangle in Figure 12a) shows the difference in the area under the curve resulting in the integral being not zero anymore. Two parameters contribute to the changing area: Known and constant period of the carrier and the slope of the modulating signal. Slope of any linear function is equal to the derivative of this function. Visible dark rectangle, or the integral taken synchronously over the full period (or multiple periods) of the excitation signal is therefore linearly related to the derivative a of the modulating signal a∙t. More precisely, the negative value of the slope of the modulating signal (-a) must be considered. It can be shown mathematically that the result is also valid for the sinusoidal excitation, which is modulated with linearly rising ramp a∙t (12) (Figure 12b):

∫ a∙x∙sin(x)dx= a∙sin(x) − a∙x∙cos (x) + C.(12)

**Figure 13:**
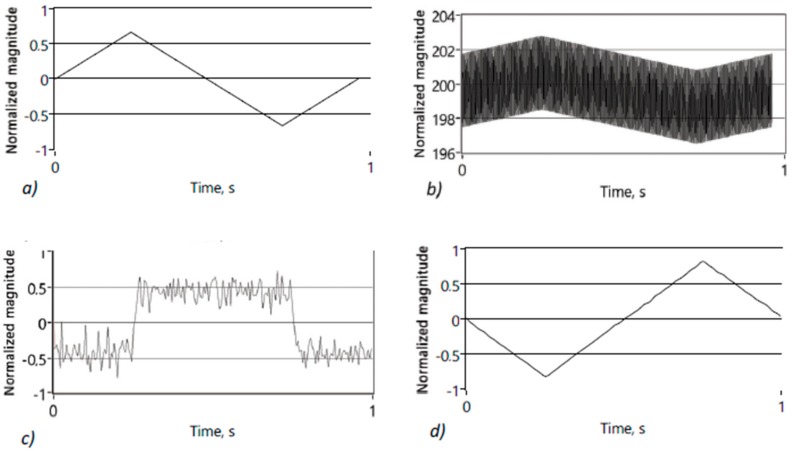
Modulating signal (**a**), signal after lock-in detector (**b**), synchronously integrated signal (**c**), signal after final integration (**d**). All signals are normalized for the sake of clarity. Please note that (**d**) is mirrored image of original (**a**), as predicted mathematically.

When Equation (12) is evaluated over the full period from 0 to 2π the result will be the same -a. If x is replaced with more typical ω∙t, where ω = 2π∙f, and f = 1/T, where ω is angular frequency in rad/s, f is frequency in 1/s or Hz, and T is period in s, then there is additional constant scale factor T^2^/2π to be considered. As the result of the synchronous integration is linearly related to the derivative of the biomodulation of the EBI it can be restored with integration if required. The main benefit of the described method is in the automatic exclusion of the base value from the result due to differentiation. Given that the signal to be digitized is now only the slow biomodulation the ADC can be slower and with fewer bits than in the previous solution. Again, the method has been extensively studied in the LabView environment with convincingly good results [26] (Figure 13).

The “derivative peak-detectors” suffer from negative side effects. The peak detection is sensitive to all components of the detectable signals, i.e., it is not selective in the frequency domain. The user of the peak detection must be aware of the unwanted additive components or noise. The situation can be improved when pre-filtering of the acquired signal is used, as it considerably reduces the impact of possible additive disturbance. Since synchronous phase sensitive measurements are required the filter should be tuned exactly without any phase variations. A typical RLC filter is not suitable here, due to large variations. Instead synchronous lock-in filtering has to be used [25]. It serves the narrow band pass filter with rock stable parameters, and as a side effect gives the base impedance value, which is useful as an indicator for the quality of the electrode attachment. Many different realizations have been proposed, such as by Märtens [27]. Both presented solutions have something in common, the need for fast synchronized polyphase switches. It is easily achievable, when using application specific integrated circuit (ASIC) realizations of the principle, but harder to achieve with standard components.

**Figure 14:**
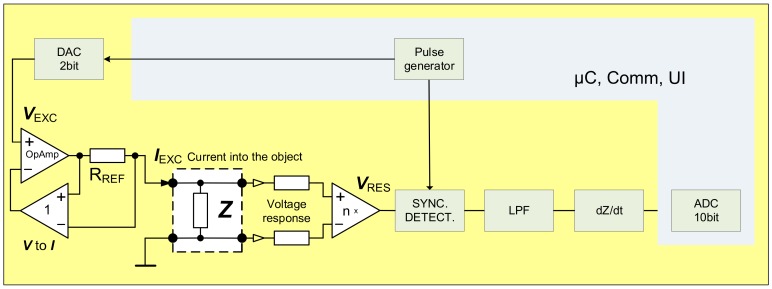
Simplified schematic of the EBI measurement device using filtering for derivation.

**Figure 15:**
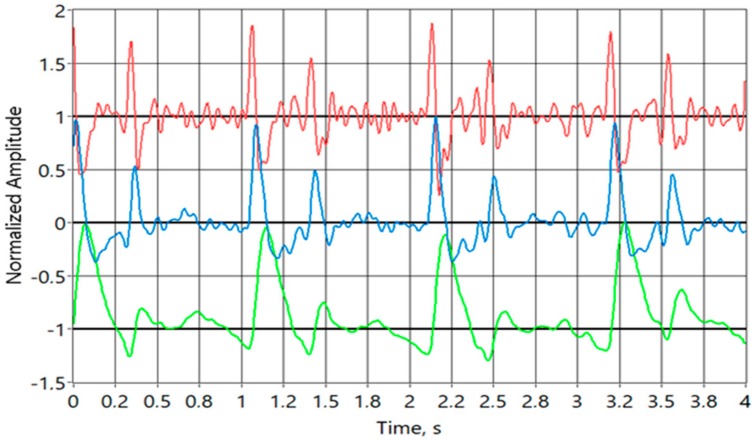
Bioimpedance signal, acquired with derivative technology, shown on Figure 11. All curves are normalized. Blue curve in the middle is the actual signal from the device, i.e., first derivative of the bioimpedance signal, upper red curve is second derivative of the bioimpedance signal shifted up by 1 unit, and lower green curve is integral of the incoming signal shifted down by 1 unit. It is also the actual bioimpedance signal.

**Figure 16:**
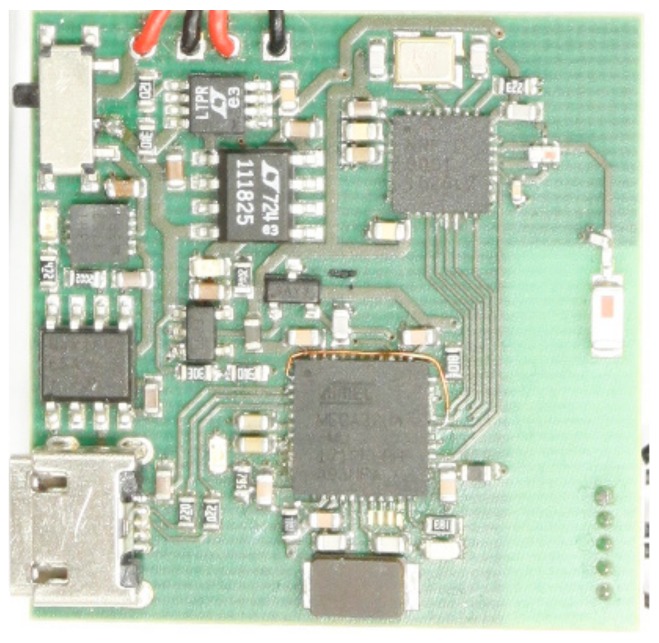
Image of the actual 10-Bit derivative bioimpedance acquisition system prototype.

The third derivative detector t is much simpler, yet performs very well (Figure 14):

The block diagram is like the solution on Figure 6. It is also similar to the solution used in [19], with one major difference. Analog derivation is used over the frequency range of interest instead of having analog low pass filter at 0.1 Hz. While, the low pass filter has an inherently long settling time, in seconds, as it has been described also in number of other publications describing similar items, the derivative approach settles in milliseconds. It is crucial when wearable implementation is used during normal everyday activity instead of static sitting or lying position. The major difference is in the block of dZ/dt. The device uses same AVR ATXMEGA microcontroller, together with BLE 4.0 + module as on Figure 6, internal rather noisy low quality 10-bit ADC was used instead of high quality external 32-bit ADC. The quality of the solution is visible on the Figure 15, representing actual waveform acquired on top of the radial artery without any additional filtering or processing. It is quite clear and a good quality signal. dZ/dt in this case is achieved with simple high pass filtering, as discussed also earlier, but with much higher corner frequency, so that in the frequency range of the bioimpedance modulation caused by the beating heart the frequency response is rising 20 dB/dec, which is effectively equal to the mathematical derivative dZ/dt. Presented derivative detector was able to resolve impedance changes as small as 4 mΩ (ADC resolution 1 mΩ/LSB), which is in the range of 1% of impedance change caused by cardiac activity. Blue curve of the Figure 15 represents derivative signal caused by impedance change of approximately 300 mΩ. Picture of the demonstrator device is visible on Figure 16.

Proposed derivative detector has several advantages compared to “Brute Force” and “Carrier Compensation” DCC method—it uses simpler hardware, has lower power consumption and requires less computational power. A comparison of the three different solutions is shown in Table 2.

## 6. Conclusions

Three derivative requiring methods for the EBI acquisition are presented. First two have been simulated in LabView environment with good results. The prototypes are being built and the last version is ready-made and tested. Differentiation implies improvement in the dynamic range of the useful signal at the input of ADC, as the variations can be 1000 times smaller than the whole measured value. Results show that the internal 10-bit analog-to-digital converter (ADC) of microcontroller is enough to achieve the signal quality, which is comparable to the one of the more complicated brute force device with 32-bit ADC’s. The difference is that the output signal is the derivative of the measured EBI. While the original EBI signal can be restored relatively easily (Figure 15), it is not mandatory as many calculations, based on the acquired signals, require derivation before further indices can be calculated.

All three derivative solutions for acquisition of the EBI signal are believed to be novel and patentable. Very good results are obtainable when used together with lock-in filtering. They are key enablers for the design of simple analog front ends for pervasive EBI acquisition with enhanced protection against artefacts. The requirements for the ADC used after detection circuitry can be substantially relaxed. The current paper is focused only on the methods and basic technology research. In terms of technology readiness levels (TRLs) it ends before TRL 3. The actual prototypes for technology demonstration belonging to TRL 5 are currently being built and tested in the frames of commercial agreement.

## Figures and Tables

**Table 1:** Impedance measurement base values, variations due to cardiac activity and phase angle.

	Z [Ω]	∆Z [Ω]	∆Z [%]	Φ [deg]
Meas. Figure 3	70.39	0.35	0.5	4.3
Reference [18]	264.57			5.6
Reference [15]	102.8	0.12	0.11	
Reference [19]		0.326		

**Table 2:** Comparison of the three different acquisition methods described.

Method	ADC Resolution	Excitation Signal Generation	Computational Power	Power Consumption	HW Complexity
DCC	16 bit	DDSx2	Medium	Hi	Hi
Brute Force	32 bit	Multibit DAC for sinusoidal signal	Hi	Medium	Medium
Derivative	10 bit	2 bit DAC for ternary signal	Low	Low	Low
